# Associations of atrial natriuretic peptide with measures of insulin and adipose depots

**DOI:** 10.14814/phy2.15625

**Published:** 2023-03-10

**Authors:** Catharine A. Couch, Lauren A. Fowler, Vibhu Parcha, Pankaj Arora, Barbara A. Gower

**Affiliations:** ^1^ Department of Nutrition Sciences University of Alabama at Birmingham Birmingham AL USA; ^2^ Division of Cardiovascular Disease University of Alabama at Birmingham Birmingham AL USA

**Keywords:** adipose tissue, insulin, natriuretic peptides, race

## Abstract

Low concentrations of natriuretic peptides (NPs) have been associated with greater risk for Type 2 diabetes (T2D). African American individuals (AA) have lower NP levels and are disproportionately burdened by T2D. The purpose of this study was to test the hypothesis that higher post‐challenge insulin in AA adults is associated with lower plasma N‐terminal pro‐atrial natriuretic peptide (NT‐proANP). A secondary purpose was to explore associations between NT‐proANP and adipose depots. Participants were 112 AA and European American (EA) adult men and women. Measures of insulin were obtained from an oral glucose tolerance test and hyperinsulinemic‐euglycemic glucose clamp. Total and regional adipose depots were measured from DXA and MRI. Multiple linear regression analysis was used to assess associations of NT‐proANP with measures of insulin and adipose depots. Lower NT‐proANP concentrations in AA participants was not independent of 30‐min insulin area under the curve (AUC). NT‐proANP was inversely associated with 30‐min insulin AUC in AA participants, and with fasting insulin and HOMA‐IR in EA participants. Thigh subcutaneous adipose tissue and perimuscular adipose tissue were positively associated with NT‐proANP in EA participants. Higher post‐challenge insulin may contribute to lower ANP concentrations in AA adults.

## Graphical abstract

NT‐proANP is inversely associated with 30‐min insulin AUC in African American (AA) participants, and with fasting insulin and HOMA‐IR in European American (EA) participants. Higher post‐challenge insulin may contribute to lower ANP concentrations in AA adults.

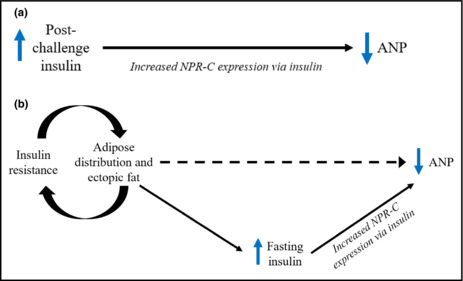

## 1 INTRODUCTION

Atrial natriuretic peptide (ANP) and brain natriuretic peptide (BNP) belong to the natriuretic peptide (NP) family of hormones with cardiovascular and renal functions, playing a key role in cardiovascular homeostasis and sodium and water balance (Kone, 2001). In addition to these essential functions, NPs possess metabolic functions including stimulation of lipolysis, adipocyte browning, and modulation of adipokine secretion (Rukavina Mikusic et al., 2022). Low circulating concentrations of NPs have been observed in individuals with obesity, insulin resistance, and Type 2 diabetes (T2D) (Khan et al., 2011; Kovacova et al., 2016; Wang et al., 2004). As a group, African American (AA) individuals have previously been shown to have lower NP levels (Bajaj et al., 2018; Gupta et al., 2015a, 2015b, 2017; Patel et al., 2019) and are disproportionately burdened by T2D and other cardiometabolic diseases (Stierman et al., 2021). Insulin, which is higher in AA individuals (Gower et al., 2002; Haffner et al., 1996), has been shown to influence NP concentrations (Bachmann et al., 2018; Pivovarova et al., 2012). It remains unclear if physiological differences in insulin action and secretion among AA and EA individuals contribute to lower NP concentrations observed in AA populations.

Lower levels of NPs have consistently been demonstrated in individuals with insulin resistance (Khan et al., 2011; Olsen et al., 2005; Wang et al., 2007) and are also associated with incident T2D (Lazo et al., 2013; Magnusson et al., 2012; Sujana et al., 2021). Additional studies that investigated the acute effects of insulin on NP concentrations observed a decrease in plasma ANP concentrations in response to an infusion of insulin (Bachmann et al., 2018; Pivovarova et al., 2012). While the mechanisms responsible for the association between insulin and NPs remain unclear, it is known that insulin upregulates expression of the NP clearance receptor (NPR‐C), thereby leading to greater clearance of NPs from circulation (Kovacova et al., 2016; Parcha et al., 2021; Pivovarova et al., 2012). AA individuals tend to have lower insulin sensitivity (Gower et al., 2002; Osei & Schuster, 1994), a higher acute insulin response to glucose (Ellis et al., 2012; Haffner et al., 1996; Osei & Schuster, 1994), and lower hepatic insulin clearance (Gower et al., 2002; Osei & Schuster, 1994), all of which act to elevate circulating insulin. Therefore, it is possible that these physiological differences in insulin secretion and action, by increasing circulating insulin, contribute to the lower levels of NPs previously observed in AA individuals (Bajaj et al., 2018; Gupta et al., 2015a, 2015b, 2017).

NPs have also emerged as regulators of lipid metabolism, exerting lipolytic functions in adipose tissue (Moro et al., 2004; Sengenès et al., 2000) and promoting browning of adipocytes (Bordicchia et al., 2012). Prior research has postulated that through these metabolic functions, NPs are capable of influencing fat distribution (Neeland et al., 2013). BNP, N‐terminal pro‐BNP (NT‐proBNP), and N‐terminal pro‐ANP (NT‐proANP) have exhibited inverse associations with BMI and visceral adiposity (Cheng et al., 2011; Sugisawa et al., 2010; Wang et al., 2004). In the Dallas Heart Study, BNP and NT‐proBNP were inversely associated with visceral fat and liver fat, and positively associated with lower body fat, independent of BMI, age, sex, and race (Neeland et al., 2013). These findings suggest that NPs may be one of the many factors involved in adipose tissue distribution.

The primary aim of this study was to test the hypothesis that higher post‐challenge insulin in AA adults is associated with lower levels of NT‐proANP. A secondary aim was to explore associations between NT‐proANP and adipose depots.

## 2 METHODS

### 2.1 Subjects and study design

This was a secondary analysis of a cross‐sectional, observational study conducted at the University of Alabama at Birmingham (UAB), between 2013 and 2018. Participants were healthy AA and EA men and women aged 19–45 years who were recruited by public advertisement (flyers and newspaper ads). Race/ancestry was determined by self‐report, and by genetic admixture analysis as described below. Recruited individuals were screened for glucose tolerance status with a 2‐h 75 g oral glucose tolerance test (OGTT), and those with 2‐h glucose ≥200 mg/dL were excluded from participation. Other exclusion criteria were absence of regular menstrual cycle; pregnant, lactating, or postmenopausal; smoking; not weight stable (change in weight > 2.5 kg in the previous 6 months); taking oral contraceptives; use of any medication known to affect carbohydrate or lipid metabolism, or energy expenditure; and use of anti‐hypertensive agents that affect glucose tolerance (e.g., thiazide diuretics at doses >25 mg/day, angiotensin‐converting‐enzyme inhibitors). Participants were instructed to maintain their usual activity level, avoid strenuous physical activity the day prior to testing, and avoid all physical activity on the morning of testing. Women were tested 3–7 days after cessation of menstruation, while in the follicular phase of the menstrual cycle. All study assessments were conducted at the core facilities of the Center for Clinical and Translational Science (CCTS), Nutrition Obesity Research Center (NORC), and Diabetes Research Center (DRC). The UAB Institutional Review Board approved the study.

### 2.2 Anthropometrics and blood pressure

Each participant underwent standard anthropometric measurements (weight and height) while wearing light clothing and no shoes. Body mass index (BMI) was calculated as weight in kilograms divided by height in meters squared (kg/m^2^). Systolic blood pressure (SBP) and diastolic blood pressure (DBP) were measured by trained nurses in the UAB Clinical Research Unit after 10 min of seated rest.

### 2.3 Oral glucose tolerance test (OGTT), insulin concentration, and insulin sensitivity

A 2‐h 75 g OGTT was completed. Participants arrived in the fasted state and venous access was obtained. Blood samples were collected at −10 min and −5 min relative to glucose load ingestion. Fasting values were calculated as the average of these two measures. A 75‐g oral glucose load was administered at time 0 and participants had 5 min to consume it. Blood samples were obtained at 10, 20, 30, 60, 90, and 120 min after glucose ingestion. Samples were processed for serum and stored at −85°C until assayed for glucose, insulin, and C‐peptide. Homeostasis model of insulin resistance (HOMA‐IR) was calculated as a measure of insulin resistance: HOMA‐IR = $\begin{array}{c} \left\{\left[\text{fasting insulin}\;\left(\mathrm{\mu U}/\mathrm{mL}\right)\right]\times \right. \end{array}$ $\begin{array}{c} \left.\left[\text{fasting glucose}\;\left(\text{mmol}/L\right)\right]\right\}/22.5.\; \end{array}$ Matsuda index was calculated as a measure of whole‐body insulin sensitivity: Matsuda $\begin{array}{c} =10,000/\surd \left[\left(G_{\text{fasting}}\times I_{\text{fasting}}\right)\times \left(G_{\text{OGTTmean}}\times I_{\text{OGTTmean}}\right)\right]\; \end{array}$, where fasting glucose and insulin are taken from time 0 of the OGTT and mean data represent the average glucose and insulin obtained during the entire OGTT (Matsuda & DeFronzo, 1999; Muniyappa et al., 2008). Total area under the curve (AUC) for insulin at 30 min (time 0 to 30 min) and 2‐h (time 0 to 120 min) were calculated using the trapezoidal rule (Tai, 1994).

### 2.4 Hyperinsulinemic‐euglycemic glucose clamp

Skeletal muscle insulin sensitivity (indicated as SI_Clamp_ for the present study) was measured using the hyperinsulinemic‐euglycemic glucose clamp. Procedures were conducted at the UAB Clinical Research Unit following a ≥10 h fast. With the participant in a recumbent position, an intravenous catheter was placed in an antecubital vein for insulin and glucose infusion. A second catheter was placed in the contralateral arm for sequential blood sampling every 10 min to determine glucose and insulin concentrations. The insulin solution (regular Humulin, Eli Lilly & Co.) was prepared with normal saline and infused at 120 mU/m^2^/min (individualized to the participant's body surface area) for 3 h using an Alaris PC unit with Guardrails software (Carefusion Corp.). Blood glucose concentrations were measured at 5 min intervals using a glucose analyzer (YSI 2300 STAT Plus, YSI, Inc.), while an infusion of 20% dextrose was adjusted to maintain blood glucose concentration at the participant's fasting level. Steady state for each individual was defined as a ≥30 min period that occurred ≥1 h after initiation of the insulin infusion, during which time the coefficients of variation for blood glucose, serum insulin, and glucose infusion rate were less than 5%. SI_Clamp_ (10^−4^.dL.kg^−1^.min^−1^/(μU/mL)) was defined as M/(G × ΔI), where M is steady state glucose infusion rate (mg/kg body mass/min), G is steady state serum glucose concentration (mg/dL), and ΔI is the difference between basal and steady state serum insulin concentrations (μU/mL). SI_Clamp_ was adjusted for total lean body mass, which was measured by dual‐energy X‐ray absorptiometry (DXA; iDXA instrument, GE Healthcare, Lunar) (Tay et al., 2020).

### 2.5 Assays

Serum concentrations of glucose, total cholesterol, high density lipoprotein cholesterol (HDL‐C), and triglycerides were measured using a Stanbio SIRRUS analyzer (Boerne, TX). Low density lipoprotein cholesterol (LDL‐C) was calculated using the Friedewald formula (Friedewald et al., 1972; Sathiyakumar et al., 2020). Insulin and C‐peptide were assayed in duplicate with a TOSOH A1A‐900 immunoassay analyzer (TOSOH Corp.). For insulin, assay sensitivity was 0.5 uU/mL, inter‐assay CV was 3.95%, and intra‐assay CV was 1.49%. Minimum assay sensitivity for C‐peptide was 0.2 ng/mL, while inter‐assay CV was 6.81% and intra‐assay was 1.67%. NT‐ProANP was measured using Human NT‐ProANP Quantikine ELISA kits (R&D Systems), with minimum assay sensitivity 0.63, intra‐assay CV 4.84%, and inter‐assay CV 4.10%.

### 2.6 Body composition

Total fat mass was measured using dual‐energy X‐ray absorptiometry (DXA; iDXA instrument, GE Healthcare, Lunar). Participants were scanned in light clothing while laying supine with arms at their sides. Abdominal subcutaneous adipose tissue (SAT), thigh SAT, intra‐abdominal adipose tissue (IAAT), thigh intermuscular adipose tissue (IMAT), thigh perimuscular adipose tissue (PMAT), liver fat, and renal sinus fat (RSF) were measured with magnetic resonance imaging (MRI) (Ingenia 1.5 T wide bore MRI system; Phillips). Liver fat was assessed using the fast spin echo 2‐point Dixon technique (Ma et al., 2005). Volumes of abdominal SAT, IAAT, and RSF were assessed via transverse abdominal images obtained via 3D volumetric T1‐weighted magnetization‐prepared rapid acquisition gradient echo (MPRAGE). The echo time, repetition time, and pulse flip angles were selected to optimize the signal‐intensity contrast between the adipose and non‐adipose tissue compartments. A series of 10 mm slices spaced at 5 mm intervals from the L1‐L4/L5 vertebrae was performed for each participant. Scans were later analyzed for volume (cm^3^) of adipose tissue using Slice‐O‐Matic image analysis software (version 4.3: Tomovision). RSF was measured from a single slice of the right and left kidneys where the fat was most visible. As the right kidney is often positioned slightly lower in the abdomen than the left kidney, a different slice was analyzed for each kidney and volume from each slice was added together for the RSF value. Thigh skeletal muscle and adipose tissue volume were analyzed using three images from the mid‐thigh (mid‐point between the anterior iliac crest and the patella). IMAT was partitioned from PMAT by manually drawing a line around the muscle itself to capture adipose tissue located directly between and within muscle groups. Scans were not available for all participants.

### 2.7 Determination of genetic admixture

Admixture analysis was performed on study participants with available DNA samples (*n* = 107). Filtering, quality control, and merging of genetic data were performed using PLINK (version 1.9) and the gaston package in R (Chang et al., 2015; Perdry et al., 2020; R Core Team, 2021). Study participants were genotyped with the Infinium Global Screening Array v3.0 (Illumina Inc.) at the Genomics Center at the University of Minnesota. CEU and YRI reference samples from 1000 Genomes Project Phase 3 were used to estimate European and African ancestry (respectively), while Colombian, Pima, Maya, Surui, and Karitiana reference samples from the Human Genome Diversity Project were used to estimate Native American ancestry (Auton et al., 2015; Cavalli‐Sforza, 2005). Prior to analysis, quality control was performed both separately for the reference and study datasets and on the merged datasets. Any individuals and variants meeting the following criteria were removed: (1) non‐autosomal SNPs; (2) SNPs or samples with call rate ≤90%; (3) SNPs deviating from Hardy–Weinberg equilibrium (*p* < 10^−7^); (4) SNPs with minor allele frequency ≤ 0.01; and (5) first‐degree relatives. Supervised admixture analysis was performed with ADMIXTURE version 1.3.0 (Alexander et al., 2009). The analysis was conducted with *K* = 3 clusters to infer ancestry fractions for individuals in the study dataset via comparison with African, European, and Native American reference populations. In AA individuals, mean African admixture was 84.64% and ranged from 67.25% to 99.99%. In EA individuals, mean African admixture was 0.59% and ranged from 0% to 6.03%. Since DNA data were missing for nine individuals, models were adjusted for self‐reported race rather than admixture to maximize sample size. In those participants with genetic admixture data, it was confirmed that results were similar regardless of whether self‐reported race or admixture was used as a covariate.

### 2.8 Statistical analyses

Group differences in descriptive characteristics were assessed by chi‐square test for categorical variables and an independent samples t‐test for continuous variables. Differences in NT‐proANP by self‐reported race were evaluated by analysis of covariance (ANCOVA) while adjusting for age, sex, BMI, and 30‐min insulin AUC. Multiple linear regression analysis was used to investigate associations of NT‐proANP with measures of insulin concentration and sensitivity (fasting insulin, 30‐min and 2‐h insulin AUC, HOMA‐IR, Matsuda index, and SI_Clamp_) with age, sex, and BMI included as covariates. ANCOVA was used to evaluate differences in adipose depots by self‐reported race while adjusting for total fat mass (abdominal SAT, thigh SAT, IAAT, liver fat, and RSF) or total fat mass and thigh skeletal muscle (IMAT and PMAT). Associations of NT‐proANP with adipose depots (abdominal SAT, thigh SAT, IAAT, liver fat, RSF, IMAT, and PMAT) were evaluated with multiple linear regression analysis, with total fat mass, age, and sex included as covariates. Models for IMAT and PMAT were additionally adjusted for thigh skeletal muscle. For linear regression models, linear relationships between the outcomes and continuous variables were confirmed with scatterplots. Residual normality was verified with histograms and QQ plots, and residual plots were examined to confirm homoscedasticity. Residuals ±3 standard deviations were considered outliers and were excluded. No evidence of multicollinearity was observed in any of the models (all variance inflation factors <5). Assumptions for all other statistical tests (data normality and equal variances) were verified prior to analysis. Any non‐normally distributed variables, including NT‐proANP, were log transformed to achieve a normal distribution. Analyses were conducted with RStudio Statistical Software (R Core Team, 2022, v4.1.3). Statistical tests were two‐tailed with significance set at *p* < 0.05.

## 3 RESULTS

A total of *N* = 112 individuals were included in the current analysis. Descriptive characteristics are presented in Table 1 by self‐reported race. The study sample was 48.2% male, with a mean ± SD age of 29.21 ± 8.06 years, and mean BMI of 27.41 ± 5.6. The median (95% CI) NT‐proANP concentration for the entire sample was 6.3 ng/mL (5.56, 7.11). BMI was significantly higher in AA participants; however, total body fat mass did not differ between the two groups. SBP was significantly higher in AA participants, while EA participants had significantly higher triglycerides. AA participants were less insulin sensitive (SI_Clamp_ and Matsuda index) and had greater 30‐min and 2‐h insulin AUC.

**TABLE 1:** Sample characteristics.

Variable	African American *n* = 54	European American *n* = 58	*p*‐value*
Age, years	30.33 ± 8.27	28.17 ± 7.79	0.16
Male/female, *n*	24/30	30/28	**0.44**
African admixture, %	84.63 ± 6.36	0.6 ± 1.12	**<0.0001**
BMI, kg/m^2^	29.21 ± 5.91	25.73 ± 4.76	**<0.001**
Total fat mass, kg	28.69 ± 12.17	24.45 ± 10.7	0.05
Total lean mass, kg	52.93 ± 11.3	48.97 ± 9.2	**<0.05**
SBP, mmHg	120.6 ± 11.4	114.2 ± 10.77	**<0.01**
DBP, mmHg	69.15 ± 9.28	67.69 ± 7.9	0.37
NT‐proANP, ng/mL	6.04 (5.3, 6.88)	6.76 (6.01, 7.6)	0.2
Cholesterol, mg/dL	168.67 (160.19, 177.6)	166.57 (157.69, 175.95)	0.74
Triglycerides, mg/dL	64.59 (56.48, 73.87)	80.64 (70.95, 91.66)	**<0.05**
LDL‐C, mg/dL	94.8 (87.4, 111)	85.2 (77.6, 95)	0.18
HDL‐C, mg/dL	57.3 ± 10.4	60.2 ± 12.1	0.18
SI_Clamp_, [10^−4^ min^−1^/(μIU/ml)]	4.53 ± 1.51	7.18 ± 1.59	**<0.0001**
HOMA‐IR	1.55 ± 1.74	1.27 ± 1.89	0.13
Matsuda index	4.89 ± 1.67	6.26 ± 1.85	**<0.05**
Fasting insulin, μIU/mL	6.97 ± 1.7	5.88 ± 1.79	0.11
30‐min Insulin AUC, μIU/mL	1077.8 (911.45, 1274.51)	822.44 (700.5, 965.57)	**<0.05**
2‐h Insulin AUC, μIU/mL	7584.9 (6588.39, 8732.15)	5632.13 (4803.34, 6603.91)	**<0.01**
Fasting glucose, mg/dL	88 ± 8.08	87.5 ± 9.54	0.75
30‐min Glucose AUC, mg/dL	3083.88 (2971.34, 3200.68)	3199.18 (3090.36, 3311.84)	0.15
2‐h Glucose AUC, mg/dL	13515.94 (12822.7, 14246.66)	13747.13 (13067.42, 14462.19)	0.64

*Note*: All values are mean ± SD or median (95% CI) unless indicated otherwise. Bold indicates statistical significance, *p* < 0.05.

Abbreviations: AUC, total area under the curve; BMI, body mass index; DBP, diastolic blood pressure; HDL‐C, high density lipoprotein cholesterol; HOMA‐IR, homeostatic model assessment for insulin resistance; LDL‐C, low density lipoprotein cholesterol; NT‐proANP, N‐terminal pro‐atrial natriuretic peptide; SBP, systolic blood pressure; SI_Clamp_, clamp insulin sensitivity.

* *p‐*value for difference between African American and European American participants.

**FIGURE 1:**
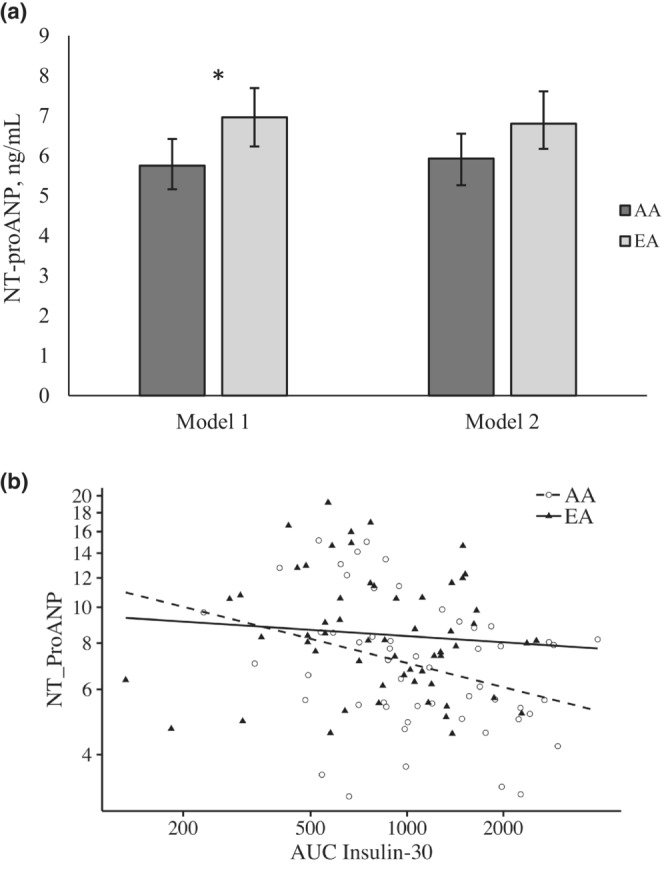
(a) NT‐proANP concentrations by self‐reported race. Data are presented as adjusted means and 95% confidence intervals. Model 1 is adjusted for age, sex, and BMI. Model 2 is adjusted for all covariates in Model 1 plus 30‐min insulin AUC. *p < 0.05. (b) Scatterplot of NT‐proANP concentrations and 30‐min insulin AUC in AA and EA participants. Data are adjusted for age, sex, and BMI. AA is African American, EA is European American.

Figure 1 shows a scatterplot of NT‐proANP concentrations and 30‐min insulin AUC, as well as ANCOVA results for differences in NT‐proANP concentrations by self‐reported race. AA participants had significantly lower NT‐proANP concentrations compared to EA participants independent of age, sex, and BMI (5.75, 95% CI [5.16, 6.42] vs. 6.96, 95% CI [6.23, 7.69] ng/mL in AA and EA, respectively; *p* = 0.02; Figure 1). Upon further adjustment for 30‐min insulin AUC, this difference was no longer significant (5.93, 95% CI [5.26, 6.55] vs. 6.9, 95% CI [6.17, 7.61] ng/mL in AA and EA, respectively; *p* = 0.07).

**FIGURE 2:**
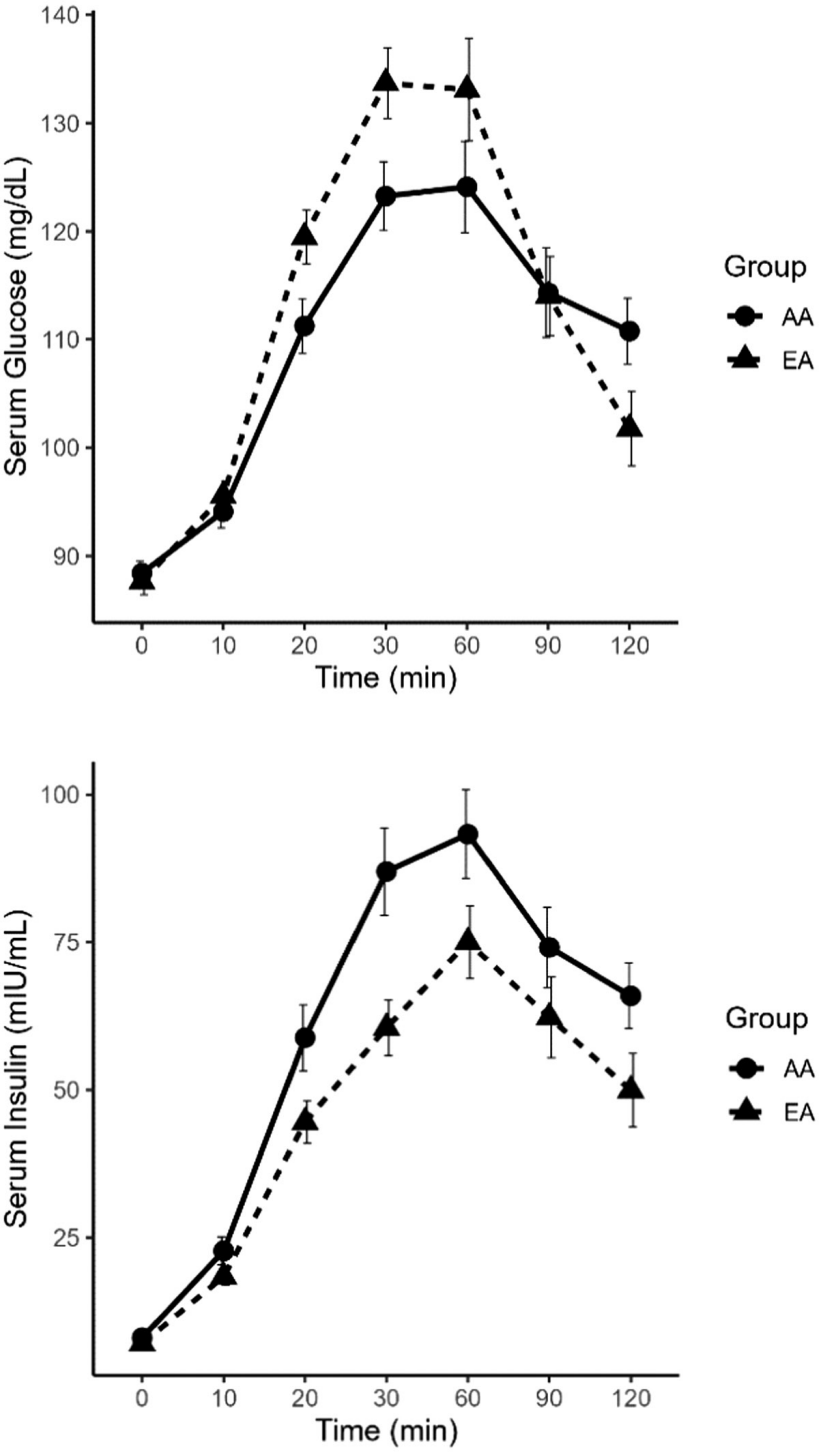
Serum glucose and insulin response to oral glucose tolerance test in African American and European American participants. Means ± SE shown. AA is African American, EA is European American.

Serum glucose and insulin responses to the OGTT are shown in Figure 2, and regression results for associations between NT‐proANP and insulin measures are reported in Table 2. In AA participants, only 30‐min insulin AUC was inversely associated with NT‐proANP concentrations in unadjusted and adjusted models. In EA participants, fasting insulin and HOMA‐IR were inversely associated with NT‐proANP concentrations in unadjusted and adjusted models. SI_Clamp_ was positively associated with NT‐proANP concentrations in EA participants; however, this association was attenuated upon further adjustment for age, sex, and BMI.

**TABLE 2:** Associations between NT‐proANP and insulin.

	African American		European American	
	Beta ± SE	*p*‐value	Beta ± SE	*p*‐value
Fasting insulin				
Model 1	−0.14 ± 0.12	0.32	**−0.29 ± 0.1**	**0.02**
Model 2	−0.04 ± 0.13	0.79	**−0.37 ± 0.1**	0.005
30‐min Insulin AUC				
Model 1	**−0.36 ± 0.1**	**0.007**	−0.11 ± 0.1	0.43
Model 2	**−0.29 ± 0.1**	**0.02**	−0.06 ± 0.09	0.61
2‐h Insulin AUC				
Model 1	−0.14 ± 0.13	0.3	−0.13 ± 0.1	0.32
Model 2	−0.1 ± 0.12	0.43	−0.12 ± 0.09	0.33
HOMA‐IR				
Model 1	−0.21 ± 0.19	0.14	**−0.29 ± 0.15**	**0.03**
Model 2	−0.11 ± 0.19	0.43	**−0.34 ± 0.15**	**0.01**
Matsuda index				
Model 1	0.22 ± 0.2	0.11	0.25 ± 0.1	0.06
Model 2	0.14 ± 0.12	0.28	0.24 ± 0.09	0.07
SI_Clamp_				
Model 1	0.09 ± 0.16	0.53	**0.36 ± 0.12**	**0.007**
Model 2	−0.13 ± 0.16	0.35	0.24 ± 0.13	0.07

*Note*: Associations were evaluated using linear regression models, with NT‐proANP as the dependent variable. Standardized betas shown. Model 1 is unadjusted, while Model 2 is adjusted for age, sex, and BMI. Bold indicates statistical significance, *p* < 0.05.

Abbreviations: AUC, area under the curve; HOMA‐IR, homeostatic model assessment for insulin resistance.

Adipose depots by self‐reported race and regression results for associations between NT‐proANP and adipose depots are shown in Tables 3 and 4. EA participants had greater IAAT volume than AA participants, and AA participants had greater IMAT and PMAT volume than EA participants. In AA participants, RSF, IMAT, and PMAT were positively associated with NT‐proANP concentrations; however, none of these associations were independent of total fat mass, age, and sex. In EA adults, thigh SAT and PMAT were positively associated with NT‐proANP concentrations independent of total fat, but not age and sex. Total fat mass was not associated with NT‐proANP in either AA or EA participants (results not shown).

**TABLE 3:** Adipose depots by self‐reported race.

Variable	African American	European American	*p*‐value*
Abdominal SAT, cm^3^	3003.04 (2777.29, 3237.61)	2851.56 (2641.96, 3069.16)	0.34
Thigh SAT, cm^3^	172.43 (152.93, 194.42)	162.39 (145.47, 181.27)	0.48
IAAT, cm^3^	343.7793 (270.43, 432.68)	502.7 (403.43, 632.7)	**0.02**
Liver fat, %	16.73 (15.13, 18.49)	18.75 (17.14, 20.52)	0.1
RSF, cm^3^	2.00 (1.8, 2.22)	1.79 (1.62, 1.99)	0.14
IMAT, cm^3^	10.18 (8.85, 11.71)	7.39 (6.49, 8.5)	**0.003**
PMAT, cm^3^	23.1 (20.91, 25.53)	17.81 (16.12, 19.69)	**0.0008**

*Note*: Data are expressed as adjusted means ± SE. Values for abdominal SAT, thigh SAT, IAAT, liver fat, and RSF are adjusted for total fat and values for IMAT and PMAT are adjusted for total fat and thigh skeletal muscle. Bold indicates statistical significance, *p* < 0.05.

Abbreviations: IAAT, intra‐abdominal adipose tissue; IMAT, intermuscular adipose tissue; PMAT, perimuscular adipose tissue; RSF, renal sinus fat; SAT, subcutaneous adipose tissue.

* *p*‐value for difference between African American and European American participants.

**TABLE 4:** Associations between NT‐proANP and adipose depots.

	African American		European American	
	Beta ± SE	*p*‐value	Beta ± SE	*p*‐value
Abdominal SAT				
Model 1	0.17 ± 0.1	0.22	0.24 ± 0.08	0.08
Model 2	−0.19 ± 0.21	0.53	0.37 ± 0.13	0.11
Model 3	−0.07 ± 0.19	0.79	0.13 ± 0.12	0.54
Thigh SAT				
Model 1	0.2 ± 0.09	0.17	**0.34 ± 0.1**	**0.01**
Model 2	0.02 ± 0.15	0.92	**0.53 ± 0.14**	**0.008**
Model 3	−0.33 ± 0.17	0.21	0.09 ± 0.17	0.71
IAAT				
Model 1	0.01 ± 0.07	0.93	−0.06 ± 0.06	0.68
Model 2	−0.18 ± 0.08	0.29	−0.21 ± 0.07	0.22
Model 3	−0.07 ± 0.11	0.78	0.11 ± 0.08	0.59
Liver fat				
Model 1	−0.24 ± 0.11	0.07	−0.08 ± 0.08	0.54
Model 2	−0.26 ± 0.2	0.05	−0.12 ± 0.08	0.39
Model 3	−0.19 ± 0.1	0.13	0.04 ± 0.07	0.7
RSF				
Model 1	**0.27 ± 0.16**	**<0.05**	−0.03 ± 0.15	0.82
Model 2	0.22 ± 0.17	0.13	−0.08 ± 0.16	0.58
Model 3	0.16 ± 0.19	0.3	−0.09 ± 0.14	0.45
IMAT^a^				
Model 1	**0.29 ± 0.08**	**0.03**	0.22 ± 0.08	0.1
Model 2	0.19 ± 0.16	0.45	0.23 ± 0.1	0.2
Model 3	0.11 ± 0.15	0.64	0.25 ± 0.09	0.12
PMAT^a^				
Model 1	**0.29 ± 0.1**	**0.03**	**0.37 ± 0.11**	**0.006**
Model 2	0.19 ± 0.17	0.4	**0.56 ± 0.16**	**0.004**
Model 3	0.15 ± 0.19	0.53	0.3 ± 0.16	0.11

*Note*: Associations were evaluated using linear regression models, with NT‐proANP as the dependent variable. Standardized betas shown. Model 1 is unadjusted. Model 2 is adjusted for total fat. Model 3 is Model 2 + age + sex. Bold indicates statistical significance, *p* < 0.05.

Abbreviations: IAAT, intra‐abdominal adipose tissue; IMAT, intermuscular adipose tissue; PMAT, perimuscular adipose tissue; RSF, renal sinus fat; SAT, subcutaneous adipose tissue.

^a^ Models also adjusted for thigh skeletal muscle.

## 4 DISCUSSION

In the present study, we sought to test the hypothesis that higher post‐challenge insulin in AA individuals would contribute to lower NT‐proANP concentrations. We observed that lower NT‐proANP concentrations in AA participants were not independent of 30‐min insulin AUC, suggesting that higher post‐challenge insulin in AA individuals likely contributes to the lower ANP observed in this population. We additionally found differential relationships of NT‐proANP with measures of insulin concentration and insulin sensitivity/resistance among AA and EA participants, suggesting that higher fasting insulin, secondary to insulin resistance, contributes to lower ANP in EA individuals. Secondary findings indicated differential associations of NT‐proANP with adipose depots in AA and EA participants, however, these associations were not independent of age and sex, suggesting that previously reported observations of associations between NPs and adipose depots were likely mediated by other factors.

Similar to previous studies, we observed AA participants to have lower NT‐proANP concentrations than EA participants (Patel et al., 2019). While it remains unclear why AA individuals have lower NP levels, it has been postulated to be partly due to differences in gene expression for enzymes involved in the processing, clearance, and regulation of NPs (Patel et al., 2019). Despite AA individuals having lower insulin sensitivity (Gower et al., 2002; Osei & Schuster, 1994), slower hepatic insulin clearance (Gower et al., 2002; Osei & Schuster, 1994), and higher acute insulin response to glucose (Ellis et al., 2012; Haffner et al., 1996; Osei & Schuster, 1994), no previous study to our knowledge has investigated the possibility that these physiological characteristics of insulin metabolism could contribute to the differences observed in NP concentrations among AA and EA individuals. We found that upon the adjustment of NT‐proANP levels for 30‐min insulin AUC, NT‐proANP levels were no longer significantly different between AA and EA participants, suggesting that the elevated acute insulin response to glucose in AA individuals contributes to the lower ANP concentrations observed in this population. NPs exert their metabolic effects by binding and activating the NP receptor A (NPR‐A) and are cleared by the NP receptor C (NPR‐C) via receptor‐mediated internalization (Kone, 2001). Insulin has been shown to downregulate NPR‐A and upregulate NPR‐C expression, with the NPR‐A‐to‐NPR‐C ratio as a major determinant of NP bioactivity (Collins, 2014; Kovacova et al., 2016). It is possible that the elevated post‐challenge insulin response in AA participants results in a lower NPR‐A‐to‐NPR‐C ratio, contributing to lower ANP concentrations. While the adjustment of NT‐proANP levels for 30‐min insulin AUC only modestly attenuated the difference between AA and EA participants, the long‐term effect of an exaggerated acute insulin response to glucose on ANP concentrations in AA individuals, and potential chronic disease risk, warrants further investigation.

Low levels of NPs are associated with insulin resistance and T2D development (Jujić et al., 2016; Khan et al., 2011; Sujana et al., 2021; Walford et al., 2014; Wang et al., 2007). Many of the studies demonstrating these associations were conducted in predominantly White populations (Jujić et al., 2016; Khan et al., 2011; Wang et al., 2007), with few including other racial/ethnic groups (Gupta et al., 2020; Walford et al., 2014), making it difficult to extend these findings to other populations. In the present study, we demonstrate unique associations between various measures of insulin and NT‐proANP in AA and EA adults. We observed NT‐proANP to be inversely associated with 30‐min insulin AUC in AA participants, potentially strengthening the observation that the acute insulin response to glucose in AA individuals contributes to racial differences in observed in NP concentrations. In EA participants, HOMA‐IR (a measure of hepatic insulin resistance) and fasting insulin were inversely associated with NT‐proANP, and SI_Clamp_ (a measure of skeletal muscle insulin sensitivity) was positively associated with NT‐proANP. It is interesting that fasting insulin was inversely associated with NT‐proANP only in EA participants. This is most likely because fasting insulin is known to be associated with insulin sensitivity, and we also observed that insulin sensitivity was associated with NT‐proANP in EA participants, but not AA participants. A recent study in the Diabetes Prevention Program (DPP) was the first to investigate whether racial differences in NPs persist over time or change in response to an intervention targeting cardiometabolic health. Results indicated that AA individuals had the lowest NT‐proBNP concentrations at baseline, and that after 2 years of follow‐up NT‐proBNP concentrations decreased only in the AA group in all study arms (placebo, lifestyle intervention, and metformin) (Gupta et al., 2020). Interventions in the DPP targeted glucose control, rather than insulin response and beta‐cell function, which could be why NP levels continued to decline in the AA group. It is also possible that differences in gene expression for enzymes involved in the processing, clearance, and regulation of NPs contributed to the continual decline of NT‐proBNP levels in the AA individuals (Patel et al., 2019). Nonetheless, the race‐specific associations observed in the present analysis indicate that while insulin acts to lower ANP in both AA and EA individuals, the mechanism behind the elevated insulin differs. In AA individuals, it is the elevated post‐challenge insulin response and in EA individuals it is insulin resistance resulting in high fasting insulin.

**FIGURE 3:**
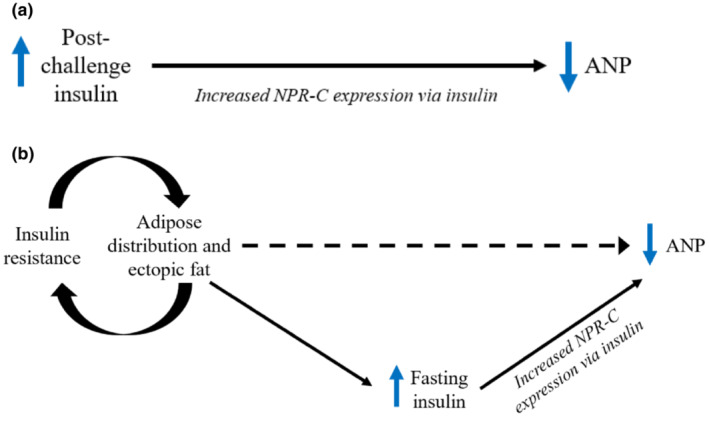
Proposed relationships among ANP, insulin, and adipose tissue in AA and EA individuals. (a) In AA adults, the elevated post‐challenge insulin response leads to an increase in NPR‐C expression resulting in increased ANP clearance and lower circulating ANP concentrations. (b) In EA adults, insulin resistance contributes to inappropriate adipose tissue expansion and ectopic fat accumulation, which leads to elevated fasting insulin. High fasting insulin increases NPR‐C expression resulting in increased ANP clearance and lower circulating ANP concentrations.

NPs have a metabolic role in human adipose tissue via lipolytic actions (Bordicchia et al., 2012; Collins, 2014). Because of this, some investigators have suggested that NPs might affect adipose tissue distribution. Only a handful of studies have investigated the relationship between NP concentrations and adipose tissue distribution, finding NP concentrations (NT‐proBNP and BNP) to be inversely associated with visceral fat and liver fat, and positively associated with lower body fat (Cheng et al., 2011; Johansen et al., 2019; Neeland et al., 2013; Sugisawa et al., 2010). These findings, along with the knowledge of NP receptors in human visceral and subcutaneous adipose tissue (Pivovarova et al., 2012), have led prior investigators to postulate that NPs influence adipose tissue distribution (Neeland et al., 2013). Results from the present study may suggest otherwise. Our results could be interpreted to suggest that insulin resistance, rather than ANP, is related to fat distribution, and that the elevated insulin that accompanies insulin resistance leads to a reduction in ANP. In EA participants we observed NT‐proANP to be inversely associated with insulin resistance and fasting insulin and positively associated with thigh SAT and PMAT. Peripheral fat expansion is positively associated with insulin sensitivity, whereas ectopic fat accumulation is associated with insulin resistance (Goedecke et al., 2009; Goss & Gower, 2012; Kelley et al., 2000). Therefore, it may be surmised that the positive association we observed of NT‐proANP with thigh SAT and PMAT suggests that greater insulin sensitivity, as reflected in greater peripheral adipose tissue and lower fasting insulin, underlies the previously observed relationship between adipose tissue distribution and NPs. Additionally, insulin resistance has been shown to have a genetic basis that relates to the inability to expand subcutaneous adipose tissue (Lotta et al., 2017). This genetic insulin resistance may explain the associations observed among NT‐proANP, insulin resistance and fasting insulin, and thigh SAT and PMAT in EA participants. Insulin resistance likely leads to elevated fasting insulin which upregulates NPR‐C and results in increased NP clearance (Kovacova et al., 2016; Pivovarova et al., 2012). Therefore, the most plausible explanation for the associations observed in the present study and previous studies, is that the association between NPs and adipose depots is driven by insulin (Figure 3a). However, the associations in our study between NT‐proANP and adipose depots were not independent of age and sex. Age and sex are known to influence insulin resistance, NP concentrations, and fat distribution, and our sample was relatively young and healthy (no T2D) compared to other studies (Cheng et al., 2011; Johansen et al., 2019; Neeland et al., 2013; Sugisawa et al., 2010). Larger studies are needed to investigate the mediating effect of insulin on the association between fat distribution and NPs, particularly in older populations with metabolic disease.

Strengths of the present study include rigorous measures of insulin sensitivity, DXA‐ and MRI‐derived measures of adipose depots, and a diverse sample. Limitations of the present study must be taken into consideration. Most importantly, as a cross‐sectional study, causality cannot be inferred. Other limitations include the secondary analysis and sample size. We also did not measure the mature peptide (ANP), which is thought to have a different clearance mechanism than the N‐terminal NP (Potter, 2011), or NP receptor expression (NPR‐A and NPR‐C). However, N‐terminal NPs and mature NPs have been demonstrated to be highly correlated (Austin et al., 2006), and thus lower NT‐proANP concentrations likely indicates lower ANP concentrations. Further, NT‐proANP was measured using fasting samples. Additional measurements of NT‐proANP following glucose challenge would have allowed for the assessment of potential associations between NT‐proANP and corresponding insulin levels. Future work will be needed to investigate the relationship between ANP and insulin over the entire course of an OGTT in AA and EA individuals. Possibly one of the greatest limitations of this work is the lack of sociocultural and environmental factors, which influence race disparities in chronic disease. It is likely that the basis of the differences observed here are due in part to the plethora of sociocultural and environmental factors that contribute to health disparities. It is also possible that these differences could be genetic or even epigenetic (potentially from sociocultural/environmental factors). Future work will be vital in identifying and understanding how all relevant factors that play a role in health disparities (i.e., genetic, epigenetic, physiological, and sociocultural) interact to contribute to differences in NT‐proANP concentrations and disease risk.

In conclusion, our results suggest that the lower NT‐proANP observed in AA individuals is related to a higher post‐challenge insulin response (Figure 3a). Insulin resistance, which is rooted in fat distribution, likely contributes to lower NT‐proANP in EA individuals via elevated fasting insulin (Figure 3b). Future studies will be needed to investigate the mediating effect of insulin on the relationship between NPs and fat distribution, and the implications of these findings for chronic disease risk in diverse populations.

## AUTHOR CONTRIBUTIONS

Catharine A. Couch and Barbara A. Gower: analysis and interpretation of data, writing – original draft; Barbara A. Gower: study design and funding acquisition; Lauren A. Fowler: genetic admixture analysis; Pankaj Arora and Vibhu Parcha: writing – review and editing. All authors reviewed and edited the manuscript and approved the final version. Catharine A. Couch and Barbara A. Gower are the guarantors of this work and, as such, had full access to all the data in the study and take responsibility for the integrity of the data and the accuracy of the data analysis.

## FUNDING INFORMATION

This study was supported by the National Institute of Diabetes and Digestive and Kidney Diseases at the National Institutes of Health (R01DK096388), UAB Nutrition Obesity Research Center (P30DK56336), UAB Diabetes Research Center (P30DK079626), and UAB CCTS Pilot Grant. CAC was supported by award number T32HL105349 by the National Heart, Lung, and Blood Institute of the National Institutes of Health.

## CONFLICT OF INTEREST STATEMENT

The authors have no conflict of interest to declare.

## ETHICS STATEMENT

This study was approved by the UAB Institutional Review Board.
